# Overall and sex-specific associations between methylation of the ABCG1 and APOE genes and ischemic stroke or other atherosclerosis-related traits in a sibling study of Chinese population

**DOI:** 10.1186/s13148-019-0784-0

**Published:** 2019-12-10

**Authors:** Xueying Qin, Jin Li, Tao Wu, Yiqun Wu, Xun Tang, Pei Gao, Lin Li, Mengying Wang, Yao Wu, Xiaowen Wang, Dafang Chen, Yonghua Hu

**Affiliations:** 1 Department of Epidemiology and Biostatistics, School of Public Health, Peking University, Beijing, 100191 China; 2 Department of Endocrinology, The PLA Rocket Force Characteristic Medical Center, Beijing, 100085 China

**Keywords:** DNA methylation, ATP-binding cassette G1, Apolipoprotein E, Ischemic stroke, Atherosclerosis

## Abstract

**Background:**

Identifying subjects with a high risk of ischemic stroke is fundamental for prevention of the disease. Both genetic and environmental risk factors contribute to ischemic stroke, but the underlying epigenetic mechanisms which mediate genetic and environmental risk effects are not fully understood. The aim of this study was to explore whether DNA methylation loci located in the ATP-binding cassette G1 (*ABCG1*) and apolipoprotein E (*APOE*) genes, both involved in the metabolism of lipids in the body, are related to ischemic stroke, using the Fangshan/Family-based Ischemic Stroke Study in China. We also tested if these CpG sites were associated with early signs of cardiovascular atherosclerosis (carotid intima–media thickness (cIMT), ankle–brachial index (ABI), and brachial–ankle pulse wave velocity (baPWV)).

**Results:**

DNA methylation at the cg02494239 locus in *ABCG1* was correlated with ischemic stroke after adjusting for gender, previous history of diabetes and hypertension, smoking, drinking, body mass index, and blood lipid levels (above vs below mean, OR = 2.416, 95% CI 1.024–5.700, *P* = 0.044; 75–100% percentile vs 0–25% percentile, OR = 4.461, 95% CI 1.226–16.225, *P* = 0.023). No statistically significant associations were observed for the cg06500161 site in *ABCG1* and the cg14123992 site in *APOE* with ischemic stroke. The study detected that hypermethylation of the *ABCG1* gene was significantly associated with cIMT, hypermethylation of the *APOE* gene was significantly related to ABI, and methylation of the *APOE* gene was statistically negatively correlated with baPWV. The above relationships demonstrated gender differences.

**Conclusions:**

These findings suggest that epigenetic modification of *ABCG1* and *APOE* may play a role in the pathway from disturbed blood lipid levels to the development of cardiovascular diseases. Future prospective validation of these findings is warranted.

## Background

DNA methylation is an epigenetic mechanism that regulates gene expression in the genome [1]. In mammals, it involves the transfer of methyl groups onto cytosine to form 5-methylcytosine. Methylation changes are related to the development and progression of a group of human diseases [2], and abnormal promotor methylation profiles have been described for several genes associated with stroke pathogenesis and recovery [3–5].

Most stroke are ischemic, and the major cause of ischemic stroke is atherosclerosis, which is a condition associated with fatty metabolic alterations or the build-up of plaques in the arteries [6, 7]. ATP-binding cassette G1 (ABCG1), one of the members of the large protein family of ABC transporters involved in the active transport of lipophilic molecules, and apolipoprotein E (APOE), the protein involved in the metabolism of lipids in the body, both affect blood lipid levels and might contribute to the development of atherosclerosis and cardiovascular disease [8, 9]. Though mutations in some genes in the lipid metabolism pathway have been well studied in ischemic stroke and atherosclerosis, increasing evidence suggests that DNA methylation in these genes plays an equally important role in ischemic stroke development and atherosclerosis. In a recent review article, Fernandez-Sanles et al. stated that differential methylation in four candidate genes, including *ABCG1*, were associated with coronary heart disease (CHD); however, *APOE* methylation was not associated with CHD. Furthermore, they noted that an epigenome-wide association study identified a set of genes, also including *ABCG1*, related to the pathogenesis of CHD, such as obesity, lipids, and inflammation [5].

Atherosclerosis is one of the primary causes of ischemic stroke and is characterized as the thickening of the arterial intima and formation of atherosclerotic lesions or atheroma, which results from the accumulation of lipids, inflammatory cells, and even vascular endothelial cells or vascular smooth muscle cells in the vessel wall. DNA methylation has been suggested to be involved in the biological processes of atherosclerosis [10].

Although both genetic and environmental risk factors contribute to ischemic stroke, the underlying epigenetic mechanisms which mediate the genetic and environmental risk effects are not fully understood. The current epigenetic studies on cardiovascular disease (CVD) have mainly been carried out in European, American, or African populations, mainly with CHD and diabetes as the outcomes. There is still insufficient evidence for the association between related gene methylation and ischemic stroke in a Chinese population.

In this study, we investigated the association of DNA methylation with ischemic stroke and related atherosclerotic traits in an established Chinese family–based population. We focused on three individual cytosine–phosphate–guanine (CpG) loci, one located in the *ABCG1* promoter region, one in the gene body region of the *ABCG1*, and the other in the *APOE* promoter region.

## Results

### Demographics of participants enrolled in the study

**Fig. 1:**
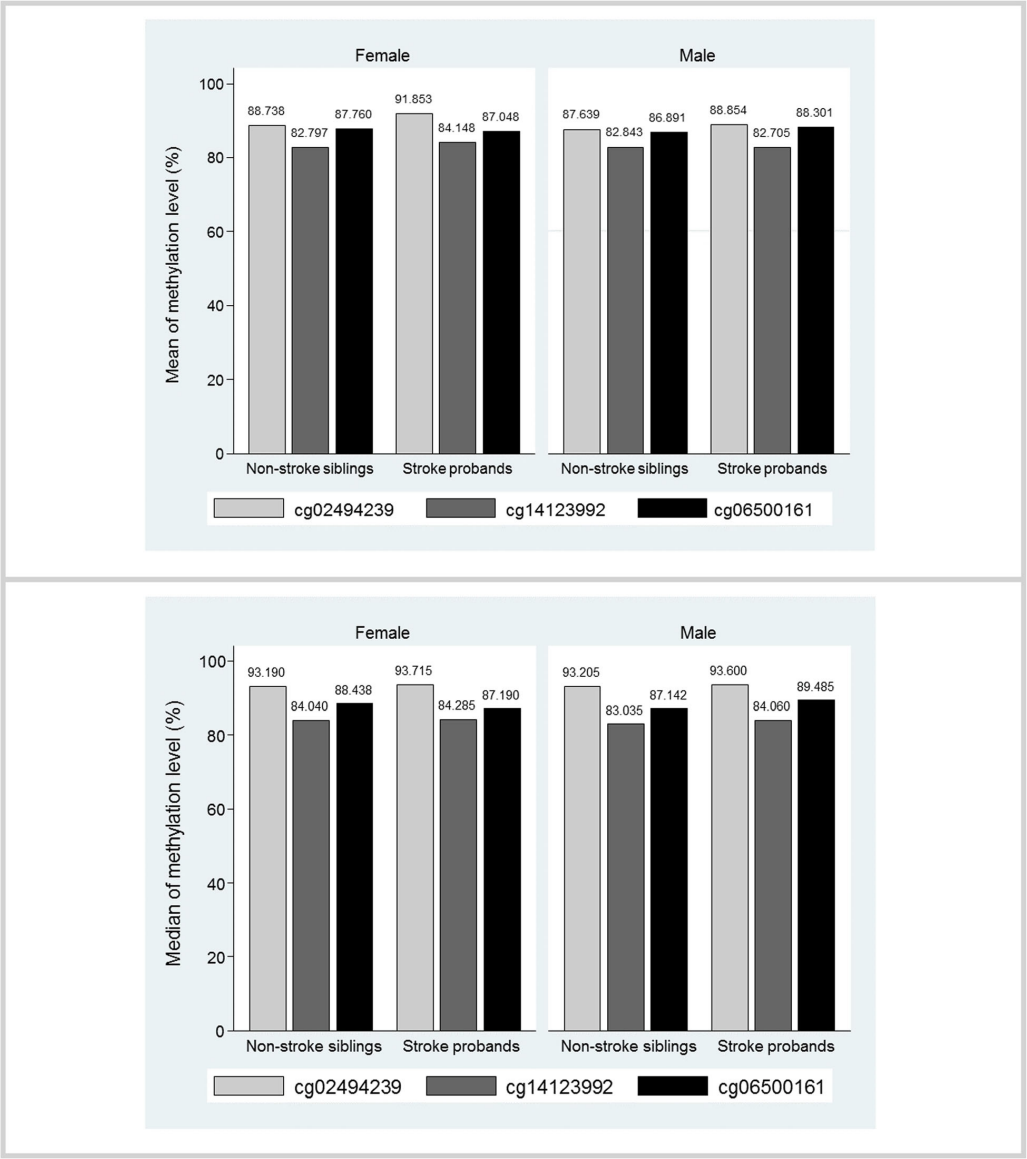
Paired comparison of *ABCG1* and *APOE* methylation levels by ischemic stroke status and sex. *ABCG1*, ATP-binding cassette G1 gene; *APOE*, apolipoprotein E gene

The distribution of characteristics for the probands and their age-matched siblings are presented in Table 1. Compared with their ischemic stroke-free siblings, the probands had a higher prevalence of diabetes (*P* = 0.020) and higher level of carotid intima–media thickness (cIMT) (*P* = 0.002), but lower level of ankle–brachial index (ABI) (*P* = 0.004). The ischemic stroke probands had similar mean and median methylation levels as their siblings at all the CpG sites, except for the median methylation level at cg02494239 (median (interquartile range): 93.660 (92.780–94.240) vs 93.260 (92.210–93.780), *P* = 0.022) (Table 1 and Fig. 1). The differences for each CpG site when comparing Q_4_, Q_3_, and Q_2_ to Q_1_ are also provided in Additional file 1: Table S1, and it showed that the cg02494239 site has larger differences in the three comparisons than the other two CpG sites.

**Table 1:** Characteristics of the study population according to ischemic stroke status

Variables	Proband (Ischemic stroke cases, *n* = 55)	Sibling (ischemic stroke-free controls, *n* = 55)	*P* value
Age (years), mean (SD)	60.055 (7.578)	59.655 (8.044)	0.262
Male (%)	37 (67.273)	30 (54.545)	0.127
Primary school or higher (%)	27 (51.923)	25 (48.077)	0.683
Marriage (%)	47 (90.385)	48 (92.308)	0.564
Smoking (%)	31 (59.615)	30 (57.692)	0.808
Drinking (%)	27 (52.941)	25 (49.020)	0.637
Diabetes (%)	20 (36.363)	11 (20.000)	0.020
Hypertension (%)	48 (87.273)	41 (74.545)	0.071
TC (mmol/L) ^*^	3.080 (2.160–3.910)	3.175 (2.270–3.720)	0.850
TG (mmol/L) ^*^	1.190 (0.770–1.950)	1.095 (0.770–1.930)	0.294
HDL (mmol/L) ^*^	0.880 (0.670–1.040)	0.840 (0.680–1.120)	0.973
LDL (mmol/L) ^*^	2.050 (1.340–2.930)	1.920 (1.510–2.680)	0.806
BMI (kg/m^2^), mean (SD)	26.531 (3.527)	26.309 (3.655)	0.694
baPWV (cm/s), mean (SD)	1779.679 (344.664)	1710.604 (328.646)	0.230
cIMT (mm), mean (SD)	0.743 (0.122)	0.695 (0.084)	0.002
ABI, mean (SD)	1.052 (0.140)	1.112 (0.087)	0.004
Methylation level (%)			
cg02494239 (*ABCG1*)			
Mean (SD)	89.835 (10.900)	88.236 (12.916)	0.111
Median (interquartile range)	93.660 (92.780–94.240)	93.260 (92.210–93.780)	0.022
Range (minimum–maximum)	46.860–95.930	40.180–95.220	
cg06500161 (*ABCG1*)			
Mean (SD)	87.891 (4.261)	87.245 (4.364)	0.292
Median (IQR)	88.475 (85.470–91.270)	88.325 (85.330–89.590)	0.436
Range (minimum–maximum)	76.460–96.040	70.080–95.635	
cg14123992 (*APOE*)			
Mean (SD)	83.177 (4.491)	82.824 (2.787)	0.635
Median (IQR)	84.200 (82.12–85.19)	83.530 (81.760–85.000)	0.105
Range (minimum–maximum)	54.450–86.660	73.140–86.400	

*TC* total cholesterol, *TG* triglyceride, *HDL* high-density lipoprotein, *LDL* low-density lipoprotein, *BMI* body mass index, *ABI* ankle–brachial index, *baPWV* brachial–ankle pulse wave velocity, *cIMT* carotid intima–media thickness, *ABI* ankle–brachial index, *ABCG1* ATP–binding cassette G1 gene, *APOE* apolipoprotein E gene, *SD* standard deviation, *IQR* interquartile range

*These quantitative variables are given as the median (interquartile range) because of their skewed distribution

After adjustment for age, gender, diabetes, and hypertension history, smoking, drinking, body mass index (BMI), and ischemic stroke status, no significant associations were found between the methylation levels at each methylation site and the lipid profiles (Additional file 1: Table S2).

### The association between ABCG1 and APOE methylation and ischemic stroke

In the overall study population, hypermethylation at cg02494239 of the *ABCG1* gene was correlated with ischemic stroke (OR = 2.416, 95% CI 1.024–5.700, *P* = 0.044), and Q_4_ group of the DNA methylation level at this CpG site was associated with a 3.461 times higher risk of ischemic stroke than Q_1_ group (OR = 4.461, 95% CI 1.226–16.225, *P* = 0.023). No statistically significant associations were observed between cg06500161 in *ABCG1* and cg14123992 in *APOE* and ischemic stroke, regardless of the types of variable. When we repeated the above analysis in men and women separately, we observed significant associations in females between the binary cg02494239 methylation level and ischemic stroke (OR = 7.941, 95% CI 1.040–60.637, *P* = 0.046), the binary cg06500161 methylation level with ischemic stroke (OR = 0.032, 95% CI 0.002–0.605, *P* = 0.022), methylation of Q_3_ group vs Q_1_ group at the cg06500161 site (OR = 0.0002, 95% CI 8.01 × 10^−8^–0.545, *P* = 0.035) and 10% increase of methylation level (OR = 0.011, 95% CI 0.0002–0.816, *P* = 0.040). In men, a higher risk of ischemic stroke was found in Q_4_ group of cg02494239 methylation in *ABCG1* compared with Q_1_ group (OR = 6.654, 95% CI 1.094–40.476, *P* = 0.040) (Table 2 and Additional file 1: Table S3).

**Table 2:** Associations between DNA methylation in *ABCG1* and *APOE* and risk of ischemic stroke

	OR	95% CI	*P* value
cg02494239			
Above vs below median	*2.416*	*1.024–5.700*	*0.044*
Q_2_ vs Q_1_	1.636	0.439–6.091	0.463
Q_3_ vs Q_1_	2.151	0.654–7.076	0.207
Q_4_ vs Q_1_	*4.461*	*1.226–16.225*	*0.023*
10% increasing of methylation level	1.254	0.875–1.795	0.217
cg06500161			
Above vs below median	0.846	0.357–2.000	0.703
Q_2_ vs Q_1_	0.641	0.183–2.243	0.486
Q_3_ vs Q_1_	0.509	0.155–1.667	0.264
Q_4_ vs Q_1_	1.000	0.291–3.441	1.000
10% increasing of methylation level	1.054	0.386–2.881	0.918
cg14123992			
Above vs below median	1.347	0.552–3.286	0.512
Q_2_ vs Q_1_	0.911	0.249–3.336	0.889
Q_3_ vs Q_1_	0.864	0.236–3.169	0.826
Q_4_ vs Q_1_	1.967	0.521–7.429	0.319
10% increasing of methylation level	0.921	0.297–2.852	0.886

Categorical variable for each CpG sites was defined using the 25% quartile, the median, and the 75% quartile of the methylation value, where Q_1_ group was 0–25% of the values, Q_2_ was 25–50%, Q_3_ was 50–75%, and Q_4_ was 75–100%. *ABCG1* ATP–binding cassette G1 gene, *APOE* apolipoprotein E gene, *OR* odds ratio, *95% CI* 95% confidence interval

### The association between ABCG1 and APOE gene methylation and cIMT, ABI, and baPWV

We observed that a higher DNA methylation level at cg02494239 in the *ABCG1* gene was associated with cIMT (above vs below median, *β* = 0.046, 95% CI 0.016–0.077, *P* = 0.003; Q_3_ group vs Q_1_ group, *β* = 0.052, 95% CI 0.008–0.097, *P* = 0.022; Q_4_ group vs Q_1_ group, *β* = 0.075, 95% CI 0.028–0.122, *P* = 0.002), and the associations remained significant in men after stratifying by gender. No significant associations were observed between the mean DNA methylation levels at the cg02494239 site and ABI and brachial–ankle pulse wave velocity (baPWV) in the whole and female populations. However, the relationships were statistically significant for this site with ABI in males (above vs below median, *β* = −0.076, 95% CI − 0.122 to − 0.029, *P* = 0.001; Q_4_ group vs Q_1_ group: *β* = − 0.076, 95% CI − 0.151 to − 0.002, *P* = 0.046) and with baPWV (Q_4_ group vs Q_1_ group: *β* = − 188.203, 95% CI − 372.297 to − 4.108, *P* = 0.045).

For the cg06500161 site, we only found significant associations for the binary variable and continuous variable of methylation level with baPWV in males (above vs below median, *β* = 161.322, 95% CI 48.604–274.041, *P* = 0.005; 10% increasing of methylation level, *β* = 182.039, 95% CI 39.992–324.087, *P* = 0.012).

**Fig. 2:**
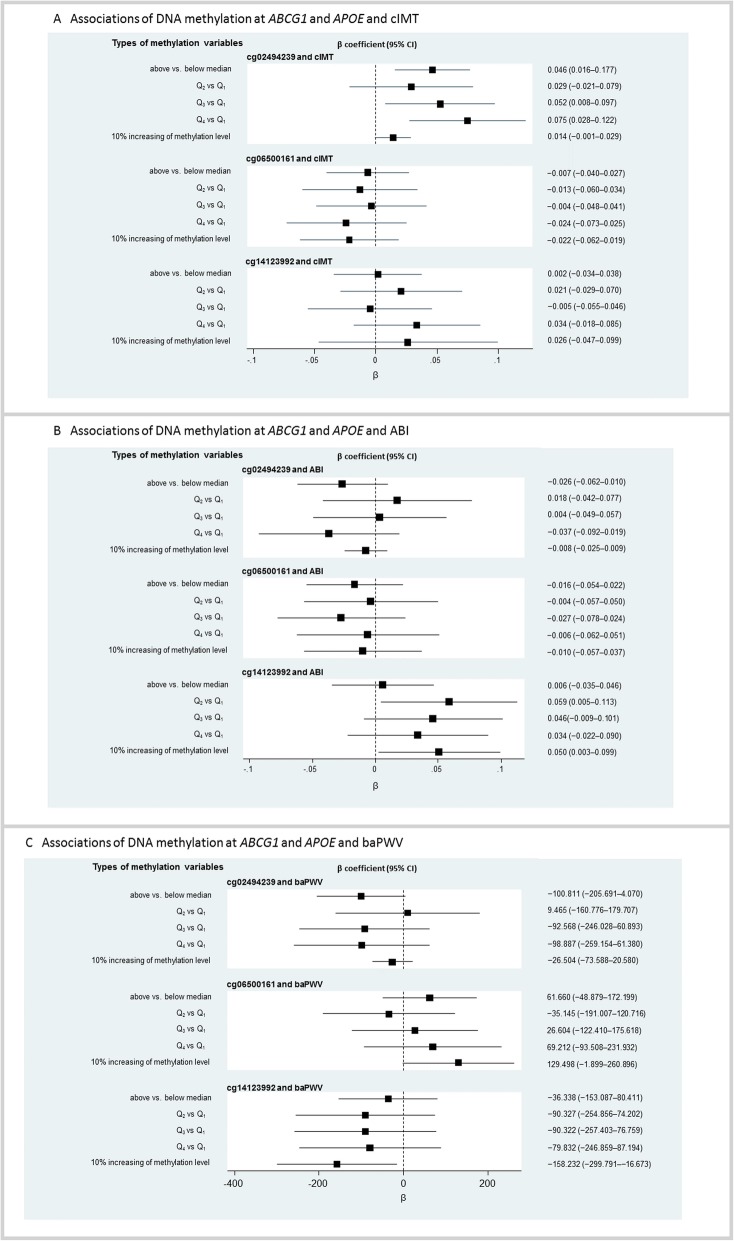
Association of DNA methylation at *ABCG1* and *APOE* and risk of atherosclerosis (cIMT, ABI, and baPWV). For a detailed analysis, methylation variable was presented as three types of variables for each CpG site, which were binary variable (above vs below median), categorical variable and continuous variable (10% increasing of methylation level). Categorical variable for each CpG sites was defined using the 25% quartile, the median, and the 75% quartile of the methylation value, where Q_1_ group was 0–25% of the values, Q_2_ was 25–50%, Q_3_ was 50–75%, and Q_4_ was 75–100%. *β*, regression coefficient; 95% CI, 95% confidence interval; *ABCG1*, ATP–binding cassette G1 gene; *APOE*, apolipoprotein E gene; cIMT, carotid intima–media thickness; ABI, ankle–brachial index; baPWV, brachial–ankle pulse wave velocity

The Q_2_ group of methylation of cg14123992 in *APOE* in the overall population was positively associated with a higher risk of ABI compared with Q_1_ group (*β* = 0.059, 95% CI 0.005–0.113, *P* = 0.033). A 10% increase in methylation level at this site was associated with a 0.050 higher ABI (95% CI 0.003–0.099, *P* = 0.039) and a 158.232 lower level of baPWV (95% CI − 299.791 to − 16.673, *P* = 0.028). Among women, the group of Q_2_, Q_3_, and Q_4_ for the *APOE* DNA methylation level was associated with cIMT compared with Q_1_ group. A higher *APOE* methylation level above the median (*β* = 0.042, 95% CI 0.007–0.076, *P* = 0.020) and Q_4_ group of the *APOE* methylation level (*β* = 0.092, 95% CI 0.032–0.152, *P* = 0.003) were associated with ABI, and a 10% increase in the methylation level was associated with a 0.093 higher ABI (*β* = 0.093, 95% CI 0.025–0.160, *P* = 0.007). A 10% increase in the *APOE* methylation level was also negatively associated with baPWV (*β* = −568.583, 95% CI − 894.388 to − 242.779, *P* = 0.001). No significant associations were observed between *APOE* gene methylation and cIMT, ABI, or baPWV in men (Fig. 2 and Additional file 1: Figure S1).

## Discussion

In this study, we evaluated the associations between DNA methylation at *ABCG1* (cg06500161 and cg02494239) and *APOE* (cg14123992) and ischemic stroke and early examinations of atherosclerosis (including cIMT, ABI, and baPWV) in subjects from a family-based study. Our results showed that a hypermethylation status in the promoter region of the *ABCG1* gene was associated with ischemic stroke, and this association was more significant in women; no associations were found between methylation levels of the *APOE* gene and ischemic stroke. The study detected that hypermethylation of the *ABCG1* gene was significantly associated with cIMT, hypermethylation of the *APOE* gene was significantly related to ABI, and the methylation of the *APOE* gene was statistically negatively correlated with baPWV; the above relationships demonstrated gender differences. cIMT and atheromatous plaques are early signs for the detection of cardiovascular atherosclerosis. ABI is important for the evaluation of peripheral atherosclerosis. baPWV is used for detecting arterial wall hardness and is closely related to cardiovascular disease. These findings suggest that DNA methylation at genes known to influence lipid levels in the body may influence the development of ischemic stroke, possibly through the lipid and fatty acid metabolism pathway.

There are few studies investigating direct associations between DNA methylation of *ABCG1* and *APOE* and ischemic stroke and atherosclerosis. Most studies focus on the association between methylation of the two genes and CHD or myocardial infarction (MI) and its major risk factors such as diabetes, obesity, and metabolic syndrome. Fernandez-Sanles et al. showed that hypermethylation in the promoter of *ABCG1* was consistently associated with CHD in candidate gene methylation studies, whereas *APOE* methylation was not [5]. Pfeiffer et al. studied the general population in Germany and found that cg06500161 in *ABCG1* was associated with HDL–C and triglyceride levels, and additionally associated with MI independent of lipid levels [11], indicating a potential role in the development of MI, which is consistent with our results. We found no association between *APOE* methylation and ischemic stroke; however, the finding from previous studies is inconsistent. Ji et al. found that *APOE* hypermethylation was significantly related to coronary heart disease in a male Chinese population [12]. Zhang et al. identified a significant association between *APOE* promoter methylation and atherosclerotic cerebral infarction in Han Chinese people after adjusting for potential risk factors such as age, gender, carotid atherosclerotic plaque, hypertension, HDL, homocysteine, and folate [13]. Ghaznavi et al. indicated that *APOE* promoter methylation status was associated with the severity of stenotic vessels in coronary artery disease in an Iranian population [14]. Karlsson et al. found no significant association between the *APOE* cg14123992 site and CVD in a Swedish twin population including 205 individuals diagnosed with CVD [15]. Different designs, population heterogeneity, sample size, and confounder adjustment may account for the inconsistency of the results from different studies.

There are gender differences in CVD mortality, and the causes for this difference are still controversial. Some studies have suggested that it may result from differences in the sex hormones or in lifestyle. Our results suggest that there is a gender difference in the association between *ABCG1* and *APOE* gene methylation and ischemic stroke and atherosclerosis, suggesting that methylation may be one of the molecular mechanisms that lead to gender differences in CVD, which is consistent with Ji et al.’s study [12]. This gender-related difference in methylation level may provide further mechanistic support for the previous findings of a greater risk of cardiovascular disease in males.

The *ABCG1* gene encodes the ABCG1 protein that is essential for the regulation of lipid metabolism and influences blood lipid levels [11, 16, 17], and the *APOE* gene is considered a candidate gene for cardiovascular disease because the APOE protein influences total serum cholesterol levels in different ways [18–21]. They both have important impact on the process of atherosclerosis [16, 22]. However, little is yet known about an epigenetic impact of *ABCG1* and *APOE* on the development of ischemic stroke. A human cell culture study showed that higher triglyceride levels in the culture media led to a reduction of macrophage ABCG1 expression, suggesting that hypermethylation in the promoter leads to downregulated expression, which could influence atherogenesis through a lower capacity for reverse cholesterol transport, thus leading to the development of atherosclerotic plaques [23]. Negative associations between *ABCG1* methylation and *ABCG1* mRNA levels, and *ABCG1* mRNA levels and HDL–C and triglyceride levels, were identified in Pfeiffer et al.’s study [11], demonstrating the potential role of DNA methylation of ABCG1 in CVD. Moreover, *ABCG1* hosts many kinds of cardio-metabolic phenotypes, including glucose and insulin measurements [24], type 2 diabetes [25, 26], and obesity [27], which are strongly related to cardiovascular diseases. Yu et al. found that one *APOE* CpG Island, which is different from the one in this study, modulates expression of the *APOE* genes and may be involved in the mechanism of action of APOE in disease risk [28]. Ma et al also identified that the methylation of CpG sites in the promoter of *APOE*, including cg14123992 and the 5′ part of the gene, were negatively associated with total cholesterol (TC), and may act as intermediate factors of the effects of age on blood lipids [29]. In order to give a deeper explanation of the role of DNA methylation on ischemic stroke and atherosclerosis based on the function of DNA methylation affecting gene expression, the mRNA expression levels should be examined to investigate the correlation between mRNA levels and methylation for each gene, but unfortunately, we were unable to detect mRNA levels for *APOE* and *ABCG1* in our subjects since the blood samples have been stored for too long time. Therefore, we referred to the method of Ma et al. [29], using published database downloaded from UCSC genome browser (methylation database: http://hgdownload.cse.ucsc.edu/goldenPath/hg19/encodeDCC/wgEncodeHaibMethyl450/; expression database: http://hgdownload.cse.ucsc.edu/goldenPath/hg19/encodeDCC/wgEncodeDukeAffyExon/) to analyze the correlation between DNA methylation and gene expression, and have updated Ma et al.’s findings. We found a significant correlation between cg14123992 and *APOE* expression (correlation coefficient = − 0.481, *P* = 0.043), which was similar to the point estimate of Ma et al [29], but differed in significance, and we found the non-significant correlation between cg06500161 and *ABCG1* expression (correlation coefficient = 0.357, *P* = 0.146). Gene expression is influenced by many factors, not just DNA methylation. Therefore, current data is very limited and complete collection of environmental, genetic, and epigenetic information will be required to ensure the accurate and reliable evidence.

In our study, we did not find significant associations between the methylation levels at each methylation site and the lipid profiles, which contradicts with the biological function of methylation that hypermethylation of these genes leads to transcriptional silencing and lowering levels of lipids. Some recent studies reported the positive associations of *APOE* and *ABCG1* methylations with lipid profiles [11, 30], but there are still some negative findings [29, 31]. Besides heterogeneity of the study populations and differences in methylation measurement methods, cross-sectional design, and small sample size in our study may partly explain the inconsistency and the non-significant associations between the methylation levels at each methylation site and the lipid profiles.

Although methylation is tissue-specific, blood sample is still a good choice for conducting epigenetic epidemiological studies for cardiovascular diseases. The symptoms of ischemic stroke are mainly characterized by impaired brain function, but the primary process of the disease itself is not generally associated with brain tissue. On the contrary, it is a disease related to vasculature, where complex interactions of lipids and endothelium occurred at the vascular and blood levels, with substantial involvement of immune cells and inflammatory factors, and ultimately leading to the formation of plaque and interrupt of blood supply to the brain [4, 32, 33]. Baccarelli et al. indicated that subjects with stroke had lower LINE-1 methylation level in blood [34], and Braun et al. found in live human individuals that the correlation coefficient of the average methylation level for each CpG across subjects was 0.86 for blood and brain, and the blood had 20.8% of CpGs that correlated to the brain, which is the highest proportion as compared to that in buccal and saliva tissues. Although this study was conducted on subjects with psychiatric diseases, it still suggested the role of blood that could be used for identifying the methylation changes associated with stroke [35]. Blood is easy to get and be tested with less invasion; therefore, it is an important tissue of ischemic stroke, and DNA methylation in blood cells is relevant for ischemic stroke and atherosclerosis.

The significance of this study on Fangshan district of Beijing is reflected in two aspects. First, Fangshan district is a district of Beijing, and the population there is a typical representative of rural Han Chinese. Fangshan district is located in the “stroke belt” of China with normally high prevalence of cardiovascular disease [36]. Second, the FISSIC study which this study was based on is established as the largest family-based study of ischemic stroke in China. Family-based samples are very valuable samples for genetic and epigenetic epidemiological studies, as it allows us to account for factors that population-based studies cannot. Based on the previous findings on the environmental and genetic risk factors of ischemic stroke in the FISSIC study, this study would provide further epigenetic evidence for ischemic stroke etiology and cardiovascular prevention in this area.

This study has some limitations. The sample size is rather small, resulting in low statistical power and unstable results. However, our samples are randomly selected from the whole sample pool, which could compensate for the impact of insufficient sample size to a certain extent. In addition, we cannot establish causal relationships between gene methylation and diseases because of the cross-sectional study design. Further prospective studies are needed. Finally, we were currently unable to measure the methylation level for more CpG sites, other epigenetic modifications, and mRNA expression levels due to the limited budget, so we cannot provide a deeper explanation for the association between epigenetic changes and ischemic stroke. But they will be the main directions for our future research.

## Conclusions

In conclusion, we found DNA methylation in *ABCG1* and *APOE* to be related to ischemic stroke and atherosclerosis in a Chinese population. Epigenetic modification of *ABCG1* and *APOE* may play a key role in the pathway from disturbed blood lipid levels to the development of cardiovascular diseases.

## Methods

### Study design and study samples

This study was based on the FISSIC study (the Fangshan/Family-based Ischemic Stroke Study in China), with details described previously [36]. Briefly, the FISSIC study is a family-based genetic pedigree study to assess the role of multiple genetic, epigenetic, and environmental risk factors involved in the etiology of ischemic stroke. We recruited ischemic stroke patients as probands and their surviving biological parents and/or siblings. The inclusion criteria for probands were: (1) confirmed ischemic stroke patients with full medical records, computerized tomography (CT), or magnetic resonance imaging (MRI); (2) older than 40 years at enrollment; (3) had at least one surviving parent or sibling who could participate in the study. Because of the late onset of ischemic stroke, most of the subjects collected were proband–sibling families. Until 2017, 2518 participants from 918 families were recruited, of which 1007 were ischemic stroke cases and 1151 were controls with no ischemic stroke.

In the current study, we employed another stricter inclusion criterion to exclude the effect of age on the outcome, so that the age difference between the proband and their siblings was no more than 2 years. Finally, 118 proband–sibling families met the above criteria, and we randomly selected 55 families from this eligible proband–sibling family pool for DNA methylation analyses, because of limited budget.

### Data collection

Data for participants included questionnaire assessments, laboratory tests, and clinical examinations.

We used a structured questionnaire to collect general demographic (such as age and gender) and lifestyle (such as smoking and alcohol-drinking habits) characteristics, and a medical history (diagnosis of hypertension and type 2 diabetes) of the subjects, through face-to-face interviews by trained investigators. Participants were categorized as smokers or non-smokers, where smokers included current smokers and former smokers. Current smokers were defined as a person who smoked at least one cigarette a day and has smoked accumulatively for 6 months or more. Former smokers were defined as people who smoked regularly in the past and have quit smoking for at least 1 month. Non-smokers were participants who had never smoked. Drinking was defined as someone who drank at least 50 ml per week of any alcohol-containing liquor for at least half a year.

Laboratory tests were done at the molecular epidemiology laboratory in the Department of Epidemiology and Biostatistics, School of Public Health, Peking University. The participants were asked not to eat after 20:00 the night before the survey. Serum blood samples were collected in EDTA tubes. All the samples were tested by auto-analyzer (Mindray BS-420; Shenzhen, China) using standard procedures. The tests included total cholesterol (TC), total triglyceride (TG), high-density lipoprotein (HDL), and low-density lipoprotein (LDL).

Clinical examinations included height, weight, cIMT, baPWV, and ABI. Height and body weight were measured by trained and certified observers using standard procedures. BMI was calculated as weight divided by height squared (kg/m^2^). Carotid ultrasound was performed by one of two trained ultrasonographers using a high-resolution B-mode real-time ultrasound system (Acuson Inc., Mountain View, CA, USA) with a probe frequency of 7.5–10.0 MHz according to study protocol. cIMT was measured using vascular research tools (VRT) 6 DEM-O software. There were three measurement sites on each side of the neck: the proximal end of the common carotid artery (CCA), the distal end of CCA, and the carotid bifurcation. Each segment was 1 cm long. The maximum value measured for each segment of blood vessels was used as the measured value, and the average of the measured values on the above six segments was taken as the cIMT value of the subject. The cIMT was determined by four trained professionals with intra-class correlation coefficients of 0.8 or higher. baPWV is a measurement of systematic arterial stiffness and ABI is valuable for screening for peripheral artery disease. Together with cIMT, they are all indicators of atherosclerotic vascular disease. The baPWV and ABI values were tested with a BP–203 RPE III automatic arteriosclerosis detection device (Omron Health Medical Co., Ltd., China). The participants were placed in a quiet position for 3 min before testing, and then the cuff was tied to both upper arm elbow joints and ankles, and the pulse wave in the brachial artery and the posterior tibial artery pulse were measured using an automated oscillometric method. baPWV was then calculated by dividing the distance between two pulse wave measurement points by the time difference between two pulse waves. The larger the value, the higher the degree of arteriosclerosis. The detector automatically calculated and recorded the baPWV value, taking the average of the left and right baPWV as the baPWV value. ABI was calculated by dividing the highest value obtained at each ankle by the highest of the arm values. The ABI of both the left and right legs was recorded, and for the definition of peripheral artery disease, the lower value of the two was considered. The methodology for baPWV and ABI measurement was the same for all participants.

### Nucleic acid extraction and measurement of the DNA methylation level

Genomic DNA was isolated from peripheral blood leukocytes with a DNA extraction kit (DP319–01; Tiangen Biotech, Beijing, China) following the manufacturer’s instructions. Bisulfite conversion was performed using the EpiTect Bisulfite Kit (QIAGEN, Germany) according to the manufacturer’s instructions. PCR of bisulfite-converted DNA samples was performed using the PyroMark PCR Kit (QIAGEN, Germany). For all assays, the amplification began with an initial activation period of 3 min at 95 °C, followed by a 3-stage cycling process of denaturation (94 °C for 30 s), annealing (56 °C for 30 s), and extension (72 °C for 1 min) for 40 cycles. The PCR process completed with a final extension period of 72 °C for 7 min. Methylation assays of the two promotor regions of *ABCG1* and *APOE* were designed with PyroMark Assay Design 2.0 (QIAGEN, Germany). The PyroMark custom assay (QIAGEN, Germany) genomic location, primer sequences, and the sequence for analysis are presented in Additional file 1: Table S4. DNA methylation was assessed using a PyroMark Q96 ID system (QIAGEN, Germany). The nucleotide dispensation order was generated by entering the sequence for analysis into the PyroMark Q96 software (QIAGEN, Germany). A non-CpG cytosine was included in the nucleotide dispensation order to detect incomplete bisulfite conversion. The methylation at each CpG site was determined using the Pyro Q-CpG software set in CpG mode.

**Fig. 3:**
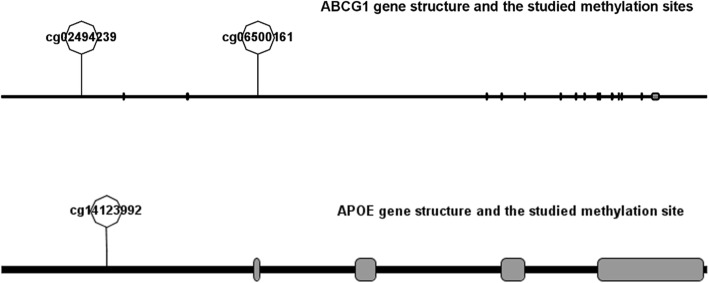
Diagram of the structure of the *ABCG1* (NCBI reference sequence: NM_207629.1) and *APOE* (NCBI Reference Sequence: NM_000041.4) genes. The line represents the gene (left to right: 5′–3′), and solid rectangles represent exons. Because of the long length of the *ABCG1* gene and the scale of the image displayed, most of the exons look like a vertical line instead of rectangles. *ABCG1*, ATP–binding cassette G1 gene; *APOE*, apolipoprotein E gene

We used candidate gene strategy to select genes and their CpG sites based on their functions and previous evidence on the association of DNA methylation, and at the same time referred to the results of BeadChip that we have conducted in a smaller population (not yet published). The cg06500161 was the most widely studied methylation site in *ABCG1*, and therefore was included in this study [24, 37, 38]. Another CpG site in *ABCG1* (cg02494239) was selected because it is located in the gene promotor area and has relatively higher differential methylation levels and *P* values between ischemic stroke cases and matched siblings than other sites located in this area, according to the BeadChip results. The cg14123992 site in *APOE* has been previously reported to be associated with late-onset disease, and therefore it was included in this study [15, 29]. The locations of the three CpG sites in the genes are shown in Fig. 3.

### Statistical analysis

Normality of the data was tested using the Shapiro–Wilk test. Continuous variables were expressed as the mean and standard deviation (SD) if normally distributed; otherwise, they were expressed as the median and interquartile range (IQR), while categorical variables were reported as frequencies and percentages (%).

First, we compared the distributions of demographic characteristics, life behaviors, medical history, BMI, and plasma lipid levels between probands and their age-matched siblings. The differences were assessed with a paired chi-square test for the qualitative variables, and a paired *t* test and non-parametric test for the normal and skewed distributed quantitative variables, respectively. We estimated the mean and the SD, the median and the IQR, the range with minimum to maximum of DNA methylation at *ABCG1* and *APOE* for probands and their siblings.

Second, we established logistic mixed-effect models for each CpG site to test whether the methylation level was associated with ischemic stroke. The general formula of the mixed-effect regression model is:

$$ Y= X\beta + Z\mu +\epsilon $$

where *Y* is a vector of the outcome variable, and *X* is a matrix of the predictor variables for the fixed effects, and *Z* is the matrix of covariates for the random effects, and ϵ is a vector of the residuals. *β* is the vector of the regression coefficient for the fixed effects, and *μ* is for the random effects. Ischemic stroke (binary variable, yes or no) was entered as a dependent variable in the logistic mixed-model, and methylation level was an independent variable as a fixed effect, and family number was entered as a random effect. Gender, previous history of diabetes and hypertension, smoking, drinking, BMI, and blood lipid levels (TC, TG, HDL, and LDL) were added as covariates to the model as fixed effects to obtain adjusted associations. For more detailed analysis, we presented methylation variable as three types of variables for each CpG site, which were binary variable, categorical variable, and continuous variable. Taking one methylation site as an example to explain how to define the different types of independent variable, we created the binary methylation variable with the median as the split point, which is higher than the median as hypermethylation and lower than the median as hypomethylation. We used the 25% quartile, the median, and the 75% quartile of the methylation value to define categorical variable, where Q_1_ group was 0–25% of the values, Q_2_ was 25–50%, Q_3_ was 50–75%, and Q_4_ was 75–100%. We defined the continuous methylation variable as the methylation value obtained in the experiment (expressed as a percentage) multiplied by 100. When the binary variable is used as the independent variable in the mixed-effect model, the hypomethylation group is used as the reference group, and when the categorical variable is used as the independent variable, Q_1_ group is used as the reference groups, and all other groups were compared with Q_1_. *β* can be obtained directly from the model, and odds ratio (OR) is the exponentiation of *β*. For the continuous methylation variable, the regression coefficient multiplied by 10 represents the degree of increase in outcome risk for every 10% increase in methylation.

Third, linear mixed-effect regression models were performed to analyze the associations between the *ABCG1* and *APOE* gene methylation levels and cIMT, ABI, and baPWV, which predict atherosclerosis. In this step, cIMT, ABI, and baPWV were the dependent variables, respectively. Definition of independent variables and covariates were the same as for the second step.

The study population was reanalyzed separately for men and women. Results were considered statistically significant when the *P* values were less than 0.05 (two sided). All statistical analyses were performed with the STATA 13.0 software (StataCorp LP, 4905 Lakeway Drive, College Station, TX77845, USA), and gene structure mapping was performed using Illustrator for Biological Sequences (IBS) software, version 1.0.3 [39].

## Supplementary information

**Additional file 1: Table S1.** The differences of methylation level (%) for each CpG site when comparing Q_4_, Q_3_ and Q_2_ to Q_1_. **Table S2.** Adjusted association between methylation level for each CpG site and blood lipid levels. **Table S3.** Sex specific associations of DNA methylation at *ABCG1* and *APOE* and risk of ischemic stroke. **Table S4.** Details of pyrosequencing assays used to determine DNA methylation. Genomic location identified using genome reference consortium human build 37 patch release 13. CpG sites are indicated in the sequence to analyze. For, forward primer; Rev, reverse primer, Seq, sequencing primer. **Figure S1.** Sex specific associations of DNA methylation at *ABCG1* and *APOE* genes and risk of atherosclerosis (cIMT, ABI and baPWV). Figure S1 Legend: For a detailed analysis, methylation variable was presented as three types of variables for each CpG site, which were binary variable (above vs below median), categorical variable and continuous variable (10% increasing of methylation level). Categorical variable for each CpG sites was defined using the 25% quartile, the median, and the 75% quartile of the methylation value, where Q_1_ group was 0–25% of the values, Q_2_ was 25–50%, Q_3_ was 50–75%, and Q_4_ was 75–100%. β: Regression coefficient; 95% CI: 95% confidence interval; *ABCG1*: ATP-binding cassette G1 gene; *APOE*: apolipoprotein E gene; cIMT: carotid intima−media thickness; ABI: ankle−brachial index; baPWV: brachial−ankle pulse wave velocity.

## Data Availability

The datasets used and/or analyzed during the current study are available from the corresponding author on reasonable request.

## Abbreviations

ABCG1: ATP-binding cassette G1
ABI: Ankle–brachial index
APOE: apolipoprotein E
baPWV: Brachial–ankle pulse wave velocity
BMI: Body mass index
cIMT: Carotid intima–media thickness
CpG: Cytosine–phosphate–guanine
CVD: Cardiovascular disease
DNA: Deoxyribonucleic acid
HDL: High-density lipoprotein
IQR: Interquartile range
LDL: Low-density lipoprotein
SD: Standard deviation
TC: Total cholesterol
TG: Triglyceride
*β*: Regression coefficient
