# Supplementary material for: Overall and sex-specific associations between methylation of the ABCG1 and APOE genes and ischemic stroke or other atherosclerosis-related traits in a sibling study of Chinese population
Source: Clin Epigenetics. 2019 Dec 10;11:189. doi: 10.1186/s13148-019-0784-0 (PMC6902418; doi:10.1186/s13148-019-0784-0)
Supplement: Supplementary file 1 — Additional file 1: Table S1. The differences of methylation level (%) for each CpG site when comparing Q4, Q3 and Q2 to Q1. Table S2. Adjusted association between methylation level for each CpG site and blood lipid levels. Table S3. Sex specific associations of DNA methylation at ABCG1 and APOE and risk of ischemic stroke. Table S4. Details of pyrosequencing assays used to determine DNA methylation. Genomic location identified using genome reference consortium human build 37 patch release 13. CpG sites are indicated in the sequence to analyze. For, forward primer; Rev, reverse primer, Seq, sequencing primer. Figure S1. Sex specific associations of DNA methylation at ABCG1 and APOE genes and risk of atherosclerosis (cIMT, ABI and baPWV). Figure S1 Legend: For a detailed analysis, methylation variable was presented as three types of variables for each CpG site, which were binary variable (above vs below median), categorical variable and continuous variable (10% increasing of methylation level). Categorical variable for each CpG sites was defined using the 25% quartile, the median, and the 75% quartile of the methylation value, where Q1 group was 0–25% of the values, Q2 was 25–50%, Q3 was 50–75%, and Q4 was 75–100%. β: Regression coefficient; 95% CI: 95% confidence interval; ABCG1: ATP-binding cassette G1 gene; APOE: apolipoprotein E gene; cIMT: carotid intima−media thickness; ABI: ankle−brachial index; baPWV: brachial−ankle pulse wave velocity. [file 13148_2019_784_MOESM1_ESM.docx]

**Additional file 1: Table S1.** The differences of methylation level (%) for each CpG site when comparing Q_4_, Q_3_ and Q_2_ to Q_1_

|  | Proband group | | |  | Sibling group | | |
| --- | --- | --- | --- | --- | --- | --- | --- |
|  | Difference | SE | 95% *CI* |  | Difference | SE | 95% *CI* |
| cg02494239 |  |  |  |  |  |  |  |
| Q_2_ vs Q_1_ | 15.610 | 3.184 | 9.218–22.002 |  | 20.038 | 3.498 | 13.015–27.061 |
| Q_3_ vs Q_1_ | 16.284 | 3.184 | 9.891–22.676 |  | 20.749 | 3.626 | 13.470–28.029 |
| Q_4_ vs Q_1_ | 17.130 | 3.245 | 10.616–23.644 |  | 21.432 | 3.626 | 14.152–28.711 |
| cg06500161 |  |  |  |  |  |  |  |
| Q_2_ vs Q_1_ | 4.576 | 0.654 | 3.263–5.890 |  | 5.320 | 0.817 | 3.679–6.960 |
| Q_3_ vs Q_1_ | 7.782 | 0.654 | 6.469–9.095 |  | 7.276 | 0.817 | 5.635–8.916 |
| Q_4_ vs Q_1_ | 10.462 | 0.667 | 9.124–11.800 |  | 10.493 | 0.833 | 8.821–12.164 |
| cg14123992 |  |  |  |  |  |  |  |
| Q_2_ vs Q_1_ | 4.656 | 1.377 | 1.891–7.422 |  | 3.595 | 0.537 | 2.518–4.673 |
| Q_3_ vs Q_1_ | 5.959 | 1.377 | 3.194–8.725 |  | 5.113 | 0.526 | 4.057–6.170 |
| Q_4_ vs Q_1_ | 7.250 | 1.404 | 4.432–10.068 |  | 6.240 | 0.537 | 5.162–7.317 |

Categorical variable for each CpG sites was defined using the 25% quartile, the median, and the 75% quartile of the methylation value, where Q_1_ group was 0%–25% of the values, Q_2_ was 25%–50%, Q_3_ was 50%–75%, and Q_4_ was 75%–100%. SE: standard error; 95% *CI*: 95% confidence interval.

**Additional file 1: Table S2. Adjusted association between methylation level for each CpG site and blood lipid levels**

|  | cg02494239 | | |  | cg06500161 | | |  | cg14123992 | | |
| --- | --- | --- | --- | --- | --- | --- | --- | --- | --- | --- | --- |
|  | β | 95% *CI* | *P* value |  | β | 95% *CI* | *P* value |  | β | 95% *CI* | *P* value |
| TC | 0.051 | −0.263–0.365 | 0.750 |  | 0.110 | −0.203–0.423 | 0.475 |  | 0.070 | −0.246–0.386 | 0.664 |
| TG | -0.047 | −0.143–0.049 | 0.339 |  | −0.001 | −0.096–0.095 | 0.986 |  | 0.019 | −0.076–0.114 | 0.695 |
| HDL | -0.041 | −0.537–0.456 | 0.872 |  | −0.043 | −0.537–0.452 | 0.866 |  | 0.188 | −0.299–0.675 | 0.450 |
| LDL | -0.026 | −0.328–0.277 | 0.867 |  | −0.092 | −0.394–0.210 | 0.550 |  | -0.262 | −0.570–0.045 | 0.095 |

The associations were adjusted for age, gender, diabetes and hypertension history, smoking, drinking, BMI and ischemic stroke status.

TC: total cholesterol; TG: triglyceride; HDL: high density lipoprotein; LDL: low density lipoprotein; 95% *CI*: 95% confidence interval

**Additional file 1: Table S3. Sex specific associations of DNA methylation at *ABCG1* and *APOE* and risk of ischemic stroke**

|  | OR | 95% *CI* | *P value* |
| --- | --- | --- | --- |
| ***Female*** |  |  |  |
| **cg02494239** |  |  |  |
| above vs below median | **7.941** | **1.040–60.637** | **0.046** |
| Q_2_ vs Q_1_ | 1.352 | 0.083–21.971 | 0.832 |
| Q_3_ vs Q_1_ | 11.703 | 0.755–181.384 | 0.079 |
| Q_4_ vs Q_1_ | 6.933 | 0.479–100.379 | 0.156 |
| 10% increasing of methylation level | 3.133 | 0.449–21.867 | 0.249 |
| **cg06500161** |  |  |  |
| above vs below median | **0.032** | **0.002–0.605** | **0.022** |
| Q_2_ vs Q_1_ | 0.029 | 0.0002–4.498 | 0.169 |
| Q_3_ vs Q_1_ | **0.0002** | **8.01×10^-8^–0.545** | **0.035** |
| Q_4_ vs Q_1_ | 0.002 | 3.37×10^-6^–1.806 | 0.074 |
| 10% increasing of methylation level | **0.011** | **0.0002–0.816** | **0.040** |
| **cg14123992** |  |  |  |
| above vs below median | 0.661 | 0.109–4.003 | 0.652 |
| Q_2_ vs Q_1_ | 18.779 | 0.129–2735.120 | 0.203 |
| Q_3_ vs Q_1_ | 2.651 | 0.038–182.871 | 0.652 |
| Q_4_ vs Q_1_ | 11.510 | 0.206–643.769 | 0.234 |
| 10% increasing of methylation level | 21.349 | 0.076–6020.948 | 0.288 |
| ***Male*** |  |  |  |
| **cg02494239** |  |  |  |
| above vs below median | 2.032 | 0.681–6.065 | 0.204 |
| Q_2_ vs Q_1_ | 1.425 | 0.269–7.533 | 0.677 |
| Q_3_ vs Q_1_ | 0.932 | 0.194–4.472 | 0.930 |
| Q_4_ vs Q_1_ | **6.654** | **1.094–40.476** | **0.040** |
| 10% increasing of methylation level | 1.090 | 0.700–1.696 | 0.703 |
| **cg06500161** |  |  |  |
| above vs below median | 2.306 | 0.735–7.240 | 0.152 |
| Q_2_ vs Q_1_ | 0.735 | 0.140–3.872 | 0.717 |
| Q_3_ vs Q_1_ | 1.783 | 0.355–8.958 | 0.483 |
| Q_4_ vs Q_1_ | 2.238 | 0.470–10.655 | 0.312 |
| 10% increasing of methylation level | 2.401 | 0.640–9.052 | 0.196 |
| **cg14123992** |  |  |  |
| above vs below median | 1.728 | 0.546–5.474 | 0.352 |
| Q_2_ vs Q_1_ | 0.568 | 0.119–2.697 | 0.476 |
| Q_3_ vs Q_1_ | 1.059 | 0.210–5.345 | 0.944 |
| Q_4_ vs Q_1_ | 1.584 | 0.308–8.155 | 0.582 |
| 10% increasing of methylation level | 0.755 | 0.211–2.697 | 0.665 |

Categorical variable for each CpG sites was defined using the 25% quartile, the median, and the 75% quartile of the methylation value, where Q_1_ group was 0%–25% of the values, Q_2_ was 25%–50%, Q_3_ was 50%–75%, and Q_4_ was 75%–100%. *ABCG1*: ATP-binding cassette G1 gene; *APOE*: apolipoprotein E gene; OR: odds ratio; 95% *CI*: 95% confidence interval.

Table S4. Details of pyrosequencing assays used to determine DNA methylation. Genomic location identified using genome reference consortium human build 37 patch release 13. CpG sites are indicated in the sequence to analyze. For, forward primer; Rev, reverse primer, Seq, sequencing primer.

| Assay ID | Gene (genomic location) | Gene region | Primer | Sequence |
| --- | --- | --- | --- | --- |
| cg02494239 | *ABCG1* (chr21: 43629989) | 5’ UTR | For: | TAGTTGAGGTTATAGGGGTATAGAGA |
|  |  |  | Rev: | CCCAAATAACTAAACAACCATTTTTAACT |
|  |  |  | Seq: | GGTTATAGGGGTATAGAGAA |
|  |  |  | Sequence to analyze: | GAAYGAGTTTGTTTAAAGAGTYGTTATTTGGTTTTGGGGTTTAGTAT |
| cg06500161 | *ABCG1* (chr21:43656587) | Body | For: | GGTTAGGAGTTTAAAAGGTTGAGTA |
|  |  |  | Rev: | AACCTAAAACCACCTCAATAAAATC |
|  |  |  | Seq: | AAATTATGTTTAAAGGAATTAGT |
|  |  |  | Sequence to analyze: | TTYGTYGGGTGTTTAGAGAAGGTTTTGGTTT |
| cg14123992 | *APOE* (chr19: 45407868) | TSS1500 | For: | TTGGGATTTATAATAGGGTTTAGGAAAGTG |
|  |  |  | Rev: | CCTCTCTAAACATCAAATTCCTTTACT |
|  |  |  | Seq: | AGGGTTTAGGAAAGTGATA |
|  |  |  | Sequence to analyze: | GYGTTTGAGYGTTTATTGTGGTTTGTTTATTGTTA |

*ABCG1*: ATP-binding cassette G1 gene; *APOE*: apolipoprotein E gene


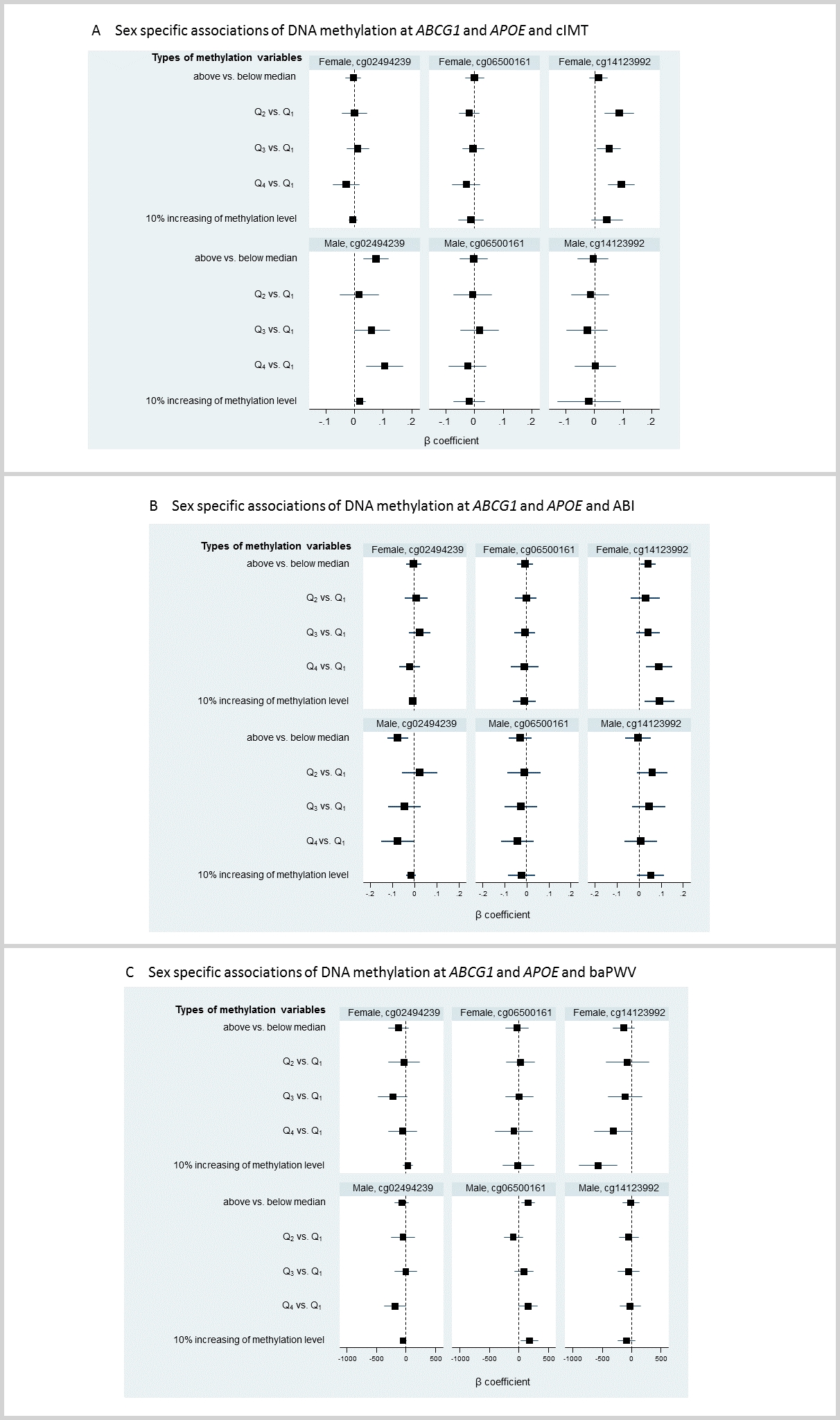


**Additional file 1: Figure S1.** Sex specific associations of DNA methylation at *ABCG1* and *APOE* genes and risk of atherosclerosis (cIMT, ABI and baPWV)

Figure S1 Legend: β: Regression coefficient; 95% *CI*: 95% confidence interval; *ABCG1*: ATP-binding cassette G1 gene; *APOE*: apolipoprotein E gene; cIMT: carotid intima−media thickness; ABI: ankle−brachial index; baPWV: brachial−ankle pulse wave velocity.
